# Matrix metalloproteinase (MMP)‐7 in Barrett's esophagus and esophageal adenocarcinoma: expression, metabolism, and functional significance

**DOI:** 10.14814/phy2.13683

**Published:** 2018-05-20

**Authors:** Hanan M. Garalla, Nantaporn Lertkowit, Laszlo Tiszlavicz, Zita Reisz, Chris Holmberg, Rob Beynon, Deborah Simpson, Akos Varga, Jothi Dinesh Kumar, Steven Dodd, David Mark Pritchard, Andrew R. Moore, András I. Rosztóczy, Tibor Wittman, Alec Simpson, Graham J. Dockray, Andrea Varro

**Affiliations:** ^1^ Institute of Translational Medicine University of Liverpool Liverpool United kingdom; ^2^ Department of Pathology University of Szeged Szeged Hungary; ^3^ Institute of Integrative Biology University of Liverpool Liverpool United kingdom; ^4^ First Department of Internal Medicine University of Szeged Szeged Hungary

**Keywords:** Barrett's esophagus, MMP‐7, myofibroblast, PI3‐kinase, proteomic

## Abstract

Matrix metalloproteinase (MMP)‐7, unlike many MMPs, is typically expressed in epithelial cells. It has been linked to epithelial responses to infection, injury, and tissue remodeling including the progression of a number of cancers. We have now examined how MMP‐7 expression changes in the progression to esophageal adenocarcinoma (EAC), and have studied mechanisms regulating its expression and its functional significance. Immunohistochemistry revealed that MMP‐7 was weakly expressed in normal squamous epithelium adjacent to EAC but was abundant in epithelial cells in both preneoplastic lesions of Barrett's esophagus and EAC particularly at the invasive front. In the stroma, putative myofibroblasts expressing MMP‐7 were abundant at the invasive front but were scarce or absent in adjacent tissue. Western blot and ELISA revealed high constitutive secretion of proMMP‐7 in an EAC cell line (OE33) that was inhibited by the phosphatidylinositol (PI) 3‐kinase inhibitor LY294002 but not by inhibitors of protein kinase C, or MAP kinase activation. There was detectable proMMP‐7 in cultured esophageal myofibroblasts but it was undetectable in media. Possible metabolism of MMP‐7 by myofibroblasts studied by proteomic analysis indicated degradation via extensive endopeptidase, followed by amino‐ and carboxpeptidase, cleavages. Myofibroblasts exhibited increased migration and invasion in response to conditioned media from OE33 cells that was reduced by MMP‐7 knockdown and immunoneutralization. Thus, MMP‑7 expression increases at the invasive front in EAC which may be partly attributable to activation of PI 3‐kinase. Secreted MMP‐7 may modify the tumor microenvironment by stimulating stromal cell migration and invasion.

## Introduction

The gastrointestinal epithelium undergoes continuous remodeling to maintain tissue architecture both in the face of normal cell proliferation and differentiation, and as part of the adaptive responses following infection or injury. The matrix metalloproteinases (MMPs) are zinc‐dependent proteases that constitute one class of extra‐cellular matrix (ECM) proteolytic enzyme that, collectively, are involved in maintaining and remodeling the ECM, and the cell attachments to it (Sternlicht and Werb 2001). These events are crucial for the orderly cell migration and invasion that underpin the maintenance of epithelial organization. Importantly, however, they are disrupted in the progression to major epithelial diseases including cancer where they participate in events central to the process of metastasis (Egeblad and Werb 2002; Gialeli et al. 2011).

The incidence of esophageal adenocarcinoma (EAC) has increased markedly in the past few decades (Pohl and Welch 2005; Zhang 2013). The most important risk factor is Barrett's esophagus (BE) which is characterized by intestinal metaplasia (IM) of the esophagus attributable to reflux of gastric acid and bile salts (Lagergren et al. 1999; Spechler and Souza 2014). The overall rate of progression from BE to EAC is relatively low with estimates of less than 0.5% of BE individuals developing EAC per annum; however, progression is increased in patients with low‐grade dysplasia (LGD) and substantially so in patients with high‐grade dysplasia (HGD) (Desai et al. 2012; Duits et al. 2015). The mechanisms that determine progression of this disease are therefore of considerable importance in developing targeted strategies for the identification, monitoring, and treatment of those patients most at risk of progression to EAC.

Matrix metalloproteinase (MMP)‐7 (also known as matrilysin or PUMP) is distinctive in that it is the smallest MMP and is predominantly expressed in epithelial cells (Wilson and Matrisian 1996). It has been linked to tissue responses to infection in the small intestine (Wilson et al. 1999), mucosal remodeling in the stomach in response to gastrin and *H.pylori* (McCaig et al. 2006; Varro et al. 2007), and in the progression of a number of epithelial cancers including stomach, pancreas, colon, and breast as well as esophagus (McDonnell et al. 1991; Adachi et al. 1998; Salmela et al. 2001; Crawford et al. 2002, 2003; Vargo‐Gogola et al. 2002). However, the mechanisms regulating expression are incompletely explored. In the stomach, there is evidence that the pyloric antral hormone gastrin regulates expression (Varro et al. 2007) and it is interesting therefore that cholecystokinin‐2 receptors (CCK2R), at which gastrin acts, are expressed in BE (Haigh et al. 2003; Lee et al. 2017). The present study was undertaken to evaluate the pattern of expression of MMP‑7 in EAC, the mechanisms regulating its expression in an EAC cell line, and its functional significance in influencing stromal cell migration and invasion.

## Materials and Methods

### Patients

Paraffin embedded tissue of surgically resected tumors, and adjacent BE or macroscopically normal tissue, from patients with EAC (*n* = 14, 9 male; 70 ± 3 years), and of endoscopic biopsies from patients with BE IM (*n* = 17, 12 male; 66 ± 3 years), low‐grade dysplasia (*n* = 17, 14 male; 65 ± 3 years) or high‐grade dysplasia (*n* = 12, 8 male; 66 ± 3) were taken from the Biobank of the Pathology Department of the University of Szeged. Histopathology was assessed by an experienced gastrointestinal pathologist using the Vienna classification (Schlemper et al. 2000; Grin and Streutker 2014). Staging of the tumors by the TNM system (Sobin et al. 2009) indicated that most were invasive, that is pT3 (pT1, 2/14; pT2, 3/14; pT3, 9/14), most exhibited no or low lymph node involvement, that is pN0 (6/14) or pN1 (6/14; pN2, 1/14, pN3, 1/14), and most were not metastatic, that is M0 (13/14; M1, 1/14). In addition, biopsies were taken for mRNA extraction from 45 patients (64 ± 2 years; 36 male) with BE IM verified by histopathology (*n* = 33, IM; *n* = 8, both IM and cardiac/fundic metaplasia; *n* = 2, IM and low‐grade dysplasia; *n* = 2, IM and adenocarcinoma) attending for routine endoscopic surveillance at the Gastrointestinal Units of the Royal Liverpool University Hospital or the First Department of Medicine of the University of Szeged. All patients gave informed consent and the studies were approved by the NHS Regional Ethics Committee or the University of Szeged ethics committee.

### Cells

The EAC and gastroesophageal junction cancer cell lines, OE33 and OE19, respectively, were obtained from American Type Culture Collection (Manassas, VA, USA); OE33‐Gr cells, expressing CCK2R, have previously been described (Haigh et al. 2003). Three cancer‐associated myofibroblast (CAM) lines generated from tumors of patients with EAC were used; these were prepared using methods previously described (Holmberg et al. 2012; Balabanova et al. 2014; Kumar et al. 2014). An earlier study showed these cells to differ in their miRNA profiles from gastric cancer and to a lesser extent squamous cell esophageal cancer myofibroblasts (Wang et al. 2016).

### Drugs and hormones

Human unsulfated heptadecapeptide gastrin (hG17) was obtained from Bachem (St Helens, Merseyside, UK). Human progastrin was a generous gift from Prof T.C. Wang (Columbia University, New York, USA), Gly‐extended gastrin‐17 (G17‐gly) and C‐terminal flanking peptide extended gastrin (G17‐CFP) were purchased from Pepsyn (Liverpool, Lancs, UK); brefeldin A (BFA), Ro32‐04332, U0126 and LY294002 were obtained from Merck Millipore (Watford, Herts, UK). Other reagents were purchased from Sigma Aldrich (Poole, Dorset, UK) unless otherwise stated.

### Immunohistochemistry

Formalin‐fixed paraffin embedded sections were processed for immunohistochemistry using antigen retrieval and stained with mouse monoclonal antibody to human MMP‑7 (Merck Millipore) as previously described (Wroblewski et al. 2003; Kumar et al. 2015). Stromal and epithelial compartments in 10 high power fields of representative sections were scored separately for staining intensity on a four‐point scale (0–3) by two independent pathologists and the percentage of stained epithelial or spindle‐shaped stromal cells at each intensity were recorded.

### Immunocytochemistry

Cultured cells were processed for immunocytochemistry using primary rabbit antibody to CCK2R (Atlas, Stockholm, Sweden) and mouse monoclonal antibody to MMP‐7 and the appropriate fluorescein labelled secondary antibody raised in donkey (Jackson Immunoresearch, Soham, Cambs, UK), and mounted with Vectashield^®^ containing DAPI (Vector Laboratories, Peterborough, Cambs, UK) as previously described (Kumar et al. 2014).

### Calcium imaging

OE33‐Gr cells were loaded with 2 *μ*mol/L Fluo‐4 AM (Invitrogen, Paisley, Renfrewshire, UK), and stimulated with hG17 (10 nmol/L) and with ionomycin (1 *μ*mol/L) as a positive control as described previously (Pennington et al. 2007; Simpson 2013).

### ELISA

Rabbit polyclonal antibodies were generated to the N‐terminal 15 residues of MMP‐7 (YSLFPNSPKWTSKVVC‐amide, corresponding to [Cys^110^]‐preproMMP‐7 95‐110) conjugated to keyhole limpet haemocyanin using m‐maleimidobenzoyl‐N‐hydroxysuccinimide ester through the C‐terminal Cys attached for the purpose (Cambridge Research Biochemicals, Billingham, Cleveland, UK). Antibodies were screened by indirect ELISA using the original antigenic peptide and L522 was selected for further analysis based on titer and specificity. An indirect ELISA was generated using Maxisorp (Thermo Scientific, Loughborough, UK) 96‐well plates, with 100 mmol/L carbonate buffer pH 9.6 for coating (2 h, on a shaker); wash buffer was 0.05% Tween‐20 in 0.02mol/L sodium barbitone buffer pH 8.4 and blocking buffer contained 1% BSA. Standards and samples were diluted in Plasma Sample Diluent (Bio‐Rad, Kidlington, Oxon, UK). Antibody L522 was used at 1:5000 (overnight, 4°C on a shaker). The secondary antibody was biotinylated anti‐rabbit IgG (Sigma Aldrich) at 1:20,000 (2 h) followed by high sensitivity streptavidin‐HRP (Thermo Scientific) at 1:20,000 and using 3,3′,5,5′ tetramethylbenzidine Supersensitive (Sigma Aldrich) as a substrate. The ELISA exhibited parallel dilution of OE33 cell extracts and media with standard recombinant human MMP‐7; the ELISA reacted with both MMP‐7 and proMMP‐7 as well as with N‐terminal fragments of MMP‐7 (95‐110 and 97‐108 of preproMMP‐7) but not with peptides corresponding to sequences C‐terminal to this region (102‐113, 114‐125, 134‐145). Inter‐assay variation was 9.1% and intra‐assay variation was 7.4%; the limit of detection was 0.015pM.

### Western blot

Media was concentrated using StrataClean resin (Agilent Technologies, Santa Clara, CA, USA) and cell extracts were prepared in RIPA buffer containing protease and phosphatase inhibitors (Merck Millipore)(Holmberg et al. 2012). Samples were resolved by SDS‐PAGE electrophoresis and processed for western blotting using antibodies to MMP‐7 (L522), MMP‐1 (R & D Systems), phospho‐ and total Akt (Cell Signaling Technology, Danvers, MA, USA). Cell extracts were reprobed for GAPDH (Biodesign, Saco, Maine, USA) to normalize for protein loading.

### Proteomic studies

Myofibroblasts were plated at 1 × 10^6^ cells in 10 cm dishes and grown for 24 h in full media. Cells were then washed three times with PBS, and cultured in serum‐free medium for 1 h. The medium was discarded and replaced with 5 mL serum‐free medium with or without MMP‐7 (2 *μ*g/mL). Medium was collected after 30 min or 24 h and centrifuged at 800*g* for 7 min. StrataClean was added to media (10 *μ*L/mL), mixed for 1 min, centrifuged and washed three times with 25 mmol/L ammonium bicarbonate. StrataClean‐bound protein samples were resuspended in 25 mmol/L ammonium bicarbonate and denatured by addition of 0.05% (w/v) RapiGest (Waters) and incubated at 80°C for 10 min. Samples were then reduced by 3 mmol/L DTT at 60°C for 10 min, alkylated by 9 mmol/L iodoacetamide for 30 min (ambient temperature in the dark) and digested by addition of sequencing‐grade trypsin in a roughly 50:1 protein:trypsin ratio (37°C, 18 h). Digested peptides were collected in a fresh tube by centrifugation at 14,000*g* for 10 min, followed by a further elution with 50 *μ*L 0.5mol/L NaCl. Samples were then desalted using C18 ZipTips (Millipore), dried, and resuspended in 20 *μ*L 3% acetonitrile, 0.1% formic acid. Samples were analysed on a Nano‐Acquity (Waters) reverse phase HPLC system in‐line with an LTQ Orbitrap Velos (Thermo).

To investigate proteolytic cleavage of MMP7, raw data files were imported into PEAKs *v*.8 (Bioinformatics Solutions Inc, ON, Canada) and de novo peptide sequence*‐*directed peak searches performed. PEAKs database searches against the Uniprot human reviewed database were carried out with no defined protease specified. Carbamidomethyl cysteine was specified as a fixed modification and oxidation of methionine as a variable modification with a precursor mass tolerance of 10 ppm and fragment ion tolerance of 0.5 Da. The data was adjusted to a peptide false discovery rate of 1%.

Raw LC‐MS files were processed using Progenesis‐QI *v*.2.0 (Waters Ltd, Newcastle‐upon‐Tyne, UK) to determine relative protein abundance. All raw files were initially automatically aligned, according to retention time, to produce an aggregate LC‐MS map, from which peptide feature charge states +1 and >+7 were excluded. Data were then separated into two experimental groups (1) 30 min MMP7 (3 biological replicates) and (2) 24 h MMP7 (3 biological replicates). An aggregate peak list file (mgf format) was then searched against the human reviewed UniProt database using the Mascot search engine (*v*. 2.4.1, Matrix Science, UK Relative protein quantification was based on averaging the individual abundances of 5 unique tryptic peptides identified for MMP7 in each sample.

### MMP‐7 knockdown

OE33 cells were transfected using Amaxa™ V cell line Nucleofector™ kit (Amaxa; Köln, Germany) with silencing RNA (siRNA) for MMP‐7 and scrambled controls (Sigma‐Aldrich) using nucleofection as previously described (Kumar et al. 2014). Control and MMP‐7 knockdown cells (10^6^ cells per flask) were then taken and further incubated for 24 h in SF medium. Conditioned medium (CM) was collected, centrifuged and stored at −80°C for migration and invasion assays.

### Cell migration and invasion assays

Transwell migration and invasion assays of myofibroblasts and OE33 cells were performed using 8 *μ*m pore size BD inserts and BD BioCoat™ Matrigel™ invasion chambers as appropriate (BD Bioscience, Oxford, UK)(Holmberg et al. 2012). Undiluted OE33 cell CM with or without MMP‐7 knockdown was placed in the well. Migrating/invading cells were fixed, stained with Quick‐Diff (Reagena, Toivala, Finland) and counted in five fields per membrane with experiments performed in triplicate. In addition, immunoneutralization using a mouse monoclonal anti‐human MMP‐7 (1:200; R & D Systems) was employed to study CAM migration in response to OE33 CM.

### Radioimmunoassay

Serum gastrin was determined by radioimmunoassay using antibody to the C‐terminus of G17 which reacts equally with G17 and G34, but not with progastrin or C‐terminal variants of G17 (Dockray et al. 1991).

### qPCR

Biopsies were extracted and RNA reverse transcribed using Promega AMV RT reagents (Kumar et al. 2015). Multiplex real time qPCR was performed using an Applied Biosystems AB7500 system with manufacturer supplied software ver2.3. The following Taqman primer/probe pairs were employed: GAPDH: 5′‐GCT CCT CCT GTT CGA CAG TCA‐3′(forward), 5′‐ACC TTC CCC ATG GTG TCT GA‐3′ (reverse), 5′‐CGT CGC CAG CCG AGC CAC A‐3′ (probe); MMP‐7: 5′‐GGA TGG TAG CAG TCT AGG GAT TAA CT‐3′ (forward), 5′‐GGA ATG TCC CAT ACC CAA AGA A‐3′ (reverse), 5′‐CCT GTA TGC TGC AAC TCA TGA ACT TGG C‐3′ (probe); CCK2R: 5′‐TGA CTC TGG GAT GCT CCT AGT‐3′ (forward), 5′‐TGG TCA GAG GTA TGA GAT TAG GC‐3′ (reverse), 5′‐ACC TCA CAG TGA CCC TTC CCA ATC AGC‐3′ (probe). Results were expressed as ΔCT normalized to GAPDH.

### Statistics

Data are expressed as mean ± SE and comparisons were made by ANOVA or *t* test as appropriate.

## Results

### Increased expression of MMP‐7 in the progression to cancer

**Figure 1:**
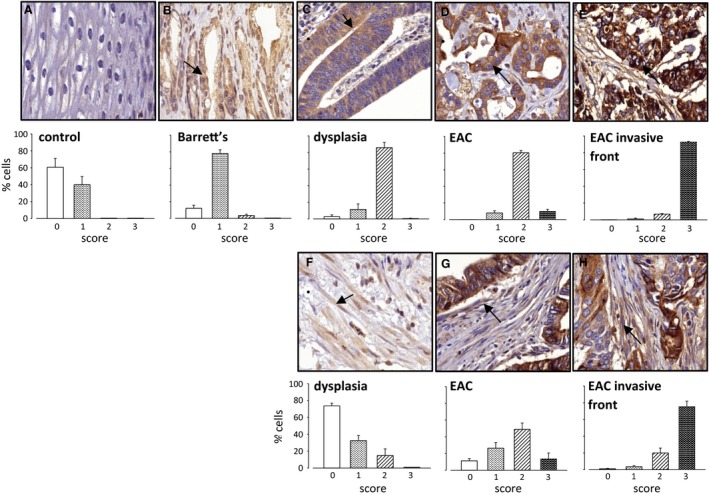
Expression of MMP‐7 in EAC and preneoplastic tissue. (A) epithelial cells in control squamous tissue (*n* = 9); (B) epithelial cells in Barrett's metaplasia (*n* = 8); (C) epithelial cells in dysplasia (*n* = 11); (D) epithelial cells in EAC (*n* = 14); (E) epithelial cells at the invasive front in EAC (*n* = 13); (F) stromal cells in dysplasia (*n* = 4); (G) stromal cells in EAC (*n* = 14); (H) stromal cells at the invasive front in EAC (*n* = 13). In each case, upper part of the panel shows a ×40 image with arrows indicating staining, and lower part shows the proportion of cells in that compartment assigned to four categories of staining intensity (0–3: 0,open bars; 1, stippling on white background; 2, cross hatching; 3, stippling on black background). Results from 14 surgically resected tumors; not all compartments were represented in all tumors, hence some “*n*” values less than 14; too few stromal cells were identified in macroscopically normal tissue, Barrett's tissue, and all but 4 of the dysplastic tissues, to be scored;. Means ± S.E.

In normal tissue adjacent to EAC tumors, MMP‐7 was expressed in <50% of squamous epithelial cells and the staining intensity was relatively low (Fig. 1A). In contrast, in Barrett's lesions adjacent to tumors there was expression in a majority of epithelial cells (78 ± 4% cells scored 1)(Fig. 1B); moreover, MMP‐7 was localized to the majority of cells in dysplastic and EAC lesions(Fig. 1C and D). For the purposes of this study, we made a distinction between staining in the body of the tumor (80 ± 3% cells scored at least 2 for intensity) and at the invasive front where over 90% scored at the highest intensity (Fig. 1D and E). Unexpectedly, in stroma putative myofibroblasts (identified as spindle‐shaped cells) were identified that expressed MMP‐7; these were scarce or absent in tissue adjacent to cancer, were present in low abundance in dysplastic regions (Fig. 1F), were commoner in the body of the tumor (Fig. 1G) and were most abundant at the invasive front (75 ± 7% cells scored 3)(Fig. 1H).

**Figure 2:**
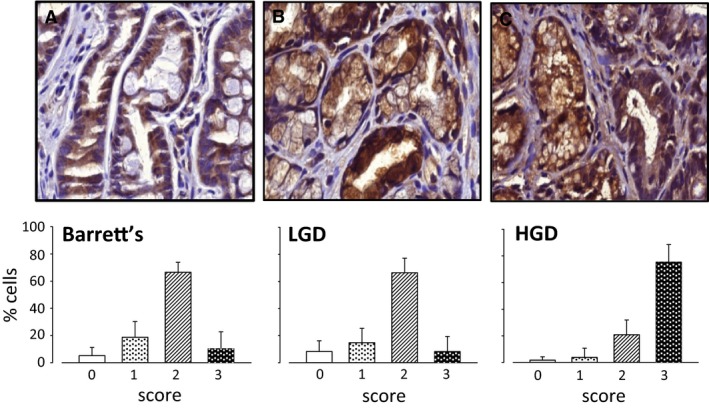
Expression of MMP‐7 in Barrett's esophagus biopsies. (A) epithelial cells in Barrett's intestinal metaplasia (*n* = 17); (B) epithelial cells in low‐grade dysplasia (*n* = 17); (C) epithelial cells in high‐grade dysplasia (*n* = 12). In each case, upper panel shows a ×40 image and the lower panel shows the proportion of cells in that compartment assigned to categories of staining intensity 0–3 (see Fig 1 legend for details). Means ± S.E.

We further examined expression of MMP‐7 in biopsies taken from BE patients exhibiting IM, LGD or HGD (Fig. 2A–C). Expression of MMP‐7 was found in virtually all epithelial cells and was at the highest intensity in HGD. In general, stromal cells were sparse in biopsies and the sample size was considered inadequate for an assessment of MMP‐7 in these cells.

### Constitutive expression of MMP‐7 in OE33 cells

**Figure 3:**
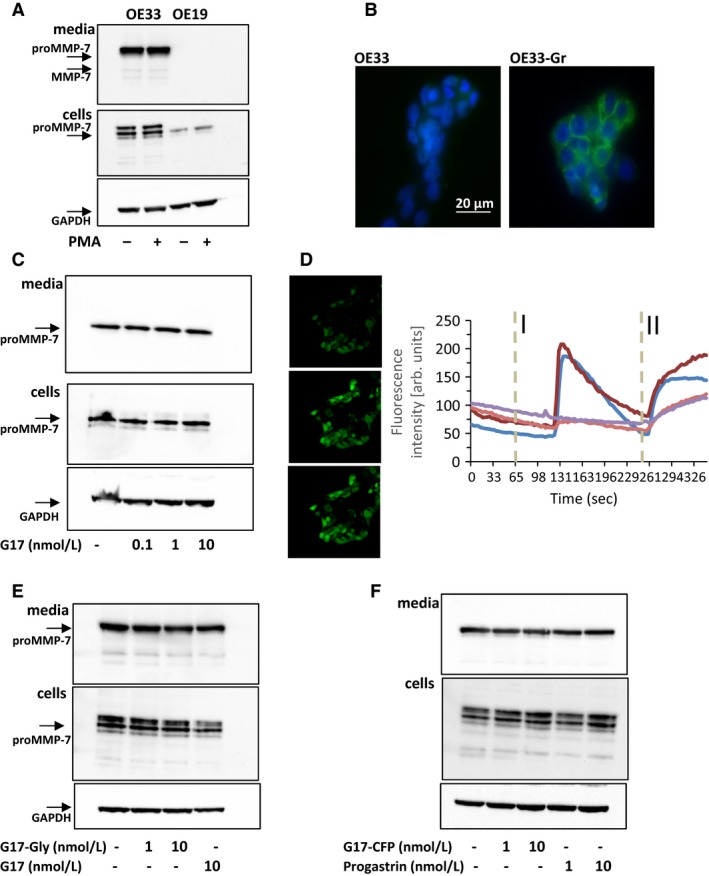
Expression of MMP‐7 in OE33 cells is insensitive to PMA and gastrin. (A) OE33, but not OE19, cell media contains abundant proMMP‐7 and a minor band corresponding to MMP‐7; there is also abundant proMMP‐7 in OE33 cell extracts and just detectable amounts in OE19 cell extracts: GAPDH in cell extracts confirms equal loading; PMA (100 nmol/L) does not stimulate proMMP‐7 expression. (B) Immunocytochemistry using CCK2R antibody confirms expression of CCK2R in OE33‐Gr cells (which have been stably transfected with CCK2R) but not wild‐type OE33 cells (green CCK2R staining; blue, nuclear staining with DAPI). (C) Heptadecapeptide gastrin (G17, 0.1–10 nmol/L) did not influence proMMP‐7 abundance in OE33‐Gr cells or media. (D) Left, representative fluorescence images of OE33‐Gr cells (top, control), treated with G17 (10 nmol/L, center), or ionomycin (bottom); right, traces from four cells, two responded to G17 (10 nmol/L, applied at I) and all responded to ionomycin (applied at II). (E) Gly‐extended G17 (G17‐Gly, 1 and 10 nmol/L) had no effect on proMMP‐7 abundance in media or cells of OE33 cells. (F) G17 extended to include the C‐terminal flanking peptide of progastrin (G17‐CFP, 1 and 10 nmol/L) and intact progastrin had no effect on proMMP‐7 abundance in OE33 media or cells.

To study in more detail the cellular basis of MMP‐7 production we first compared the EAC and gastroesophageal junction cell lines OE33 and OE19, respectively, for expression and secretion using western blot and ELISA. A band corresponding to proMMP‐7 was detectable in low abundance in OE19 cells but was undetectable in media; in contrast, in OE33 cells there was high abundance of proMMP‐7 in both cells and media and there was a minor band corresponding to mature MMP‐7 (Fig. 3A). Since BE cells express CCK2R and gastrin stimulates MMP‐7 expression in the stomach (Haigh et al. 2003; Varro et al. 2007) we then examined the effect of hG17 in OE33‐Gr cells that express CCK2R (Fig. 3B). However, in the presence of hG17 there was no change in proMMP‐7 in the media of OE33‐Gr cells (Fig. 3C). Similarly, secretion was unchanged after stimulation of protein kinase C (which is downstream of CCK2R) using phorbol 12‐myristate 13‐acetate (PMA, 100 nmol/L)(Fig. 3A) in a concentration that increased proMMP‐1 and proMMP‐3 abundance in media (not shown). Nevertheless, proof of the functionality of the receptor was established by showing increased intracellular calcium in response to hG17 (Fig. 3D). Various other forms of gastrin, for example, Gly‐extended gastrins have been reported to act on BE cells (Ogunwobi and Beales 2008; Beales and Ogunwobi 2009) but in the present study G17‐Gly, progastrin or G17‐CFP did not influence proMMP‐7 in OE33 cell media (Fig. 3E and F). The data suggest that there is high constitutive secretion of proMMP‐7 in OE33 cells.

In order to determine whether the insensitivity to gastrin seen in in vitro studies might have relevance in vivo*,* we examined MMP‐7 transcript abundance by qPCR in BE biopsies from patients with serum gastrin concentrations ranging from 6 to 495pM. There was no evidence of a correlation between MMP‐7 transcript abundance and serum gastrin concentration in this group of patients (Spearman rank correlation: *P* = 0.89, 43 degrees of freedom, *ρ*: ‐0.02). The expression of CCK2R transcripts in the same samples was verified and interestingly there was an inverse correlation between ΔCT for CCK2R normalized to GAPDH versus serum gastrin using a Spearman rank test (*P* = 0.045, 43 degrees of freedom, *ρ*: 0.300).

### Expression of proMMP‐7 in OE33 cells depends on PI 3‐kinase

**Figure 4:**
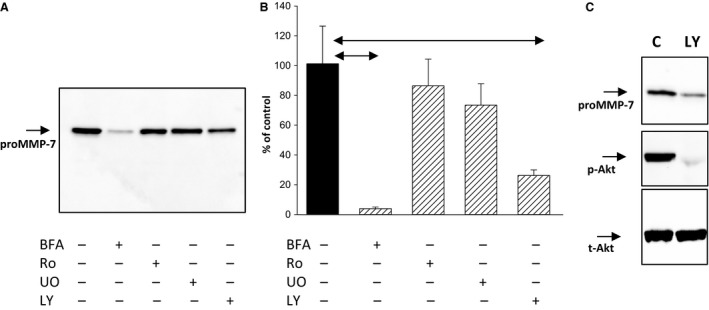
Role of PI 3‐kinase in MMP‐7 expression in OE33 cells. (A) Western blot showing suppression of proMMP‐7 in media of OE33 cells following treatment with brefeldin A (BFA, 10 *μ*g/mL) and LY294002 (LY, 50 *μ*mol/L) but not Ro32‐0432 (Ro, 2 *μ*mol/L) or UO126 (UO, 10 *μ*mol/L). (B) Similar data obtained using an ELISA for MMP‐7 in media. (C) Inhibition of PI 3‐kinase with LY294002 suppressed phosphorylation (p‐, phospho; t‐, total) of the downstream mediator Akt in cell extracts. In each case, experiments were performed in serum‐free medium added together with relevant drugs at the start of the experiment, the duration of the experiment was 6 h. Horizontal bars, *P* < 0.05.

The release of proMMP‐7 into the media by OE33 cells was shown to require integrity of the secretory pathway since it was blocked by brefeldin A (Fig. 4A and B). However, inhibitors of both protein kinase C (Ro32‐0432) and p42/44 MAP‐kinase activation (U0126) had only a small effect on proMMP‐7 in the medium indicating that secretion was unlikely to be attributable to drive from these signaling pathways (Fig 4A,B); at the concentrations used, Ro32,0432 and U0126 have been shown to virtually abolish the MMP‐1 response to PMA (Kumar et al. 2015). Interestingly, an inhibitor of phosphatidylinositol‐3‐kinase (PI 3‐kinase)(LY294002) decreased the abundance of proMMP‐7 in medium detected by western blot (Fig. 4A) and also by ELISA (Fig 4B); it also decreased abundance in cell extracts (Fig. 4C). The effect of LY294002 in suppressing PI 3‐kinase in OE33 cells was verified by demonstrating inhibition of phosphorylation of the downstream target, Akt (Fig. 4C). The data suggest, therefore, that constitutive activation of PI 3‐kinase is at least partly responsible for the high relative abundance of proMMP‐7 in OE33 cells and media.

### Expression of proMMP‐7 but not secretion in myofibroblasts

**Figure 5:**
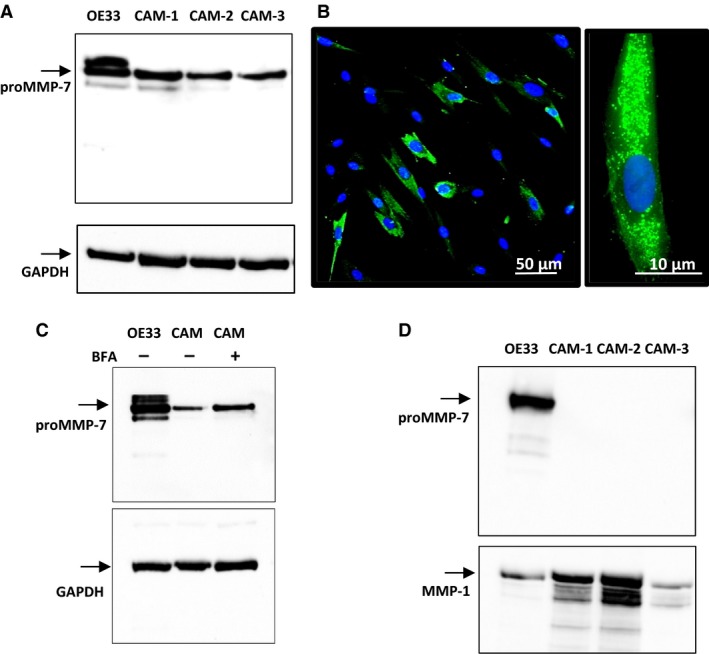
Expression of MMP‐7 in myofibroblasts. (A) In cell extracts of three different CAM lines (CAM‐1, ‐2 and ‐3) there was a band corresponding to proMMP‐7 (28 kD) in western blots; in some cases, and in OE33 cells run as a positive control, there was a minor band at 26 kD. (B) Immunocytochemistry of MMP‐7 in myofibroblasts revealed expression (green) in a high proportion of cells (left), and at a higher power (right) localization is confirmed to vesicular organelles. (C) In cell extracts of myofibroblasts treated with BFA to arrest secretion there was an increase in intracellular proMMP‐7. (D) However, in the media of three different CAM lines proMMP‐7 or related proteins were undetectable, although in the same experiments there was abundant proMMP‐7 in OE33 cell media run as a positive control, and there was abundant MMP‐1 in both CAM and OE33 cell media.

To examine in more detail the expression of MMP‐7 in myofibroblasts we screened three different esophageal adenocarcinoma‐derived CAMs. Cell extracts of all three CAM lines contained detectable proMMP‐7 (Fig. 5A) and IHC indicated punctate staining of MMP‐7 as expected for a secretory protein (Fig. 5B). Moreover, treatment of CAMs with BFA to arrest progression along the secretory pathway produced an increase in intracellular proMMP‐7 (Fig. 5C). However, unlike OE33 cells we were unable to detect proMMP‐7 in the media of myofibroblasts although there was abundant MMP‐1 in media which was used as a positive control (Fig. 5D). On the basis of ELISA data, we estimate that concentrations of proMMP‐7 activity in media equate to secretion of less than 0.25 pmol per 10^6^ myofibroblasts in 24 h compared with 5–10 pmol per 10^6^ OE33 cells in 24 h.

### Proteomic studies show degradation of MMP‐7 by myofibroblasts

**Figure 6:**
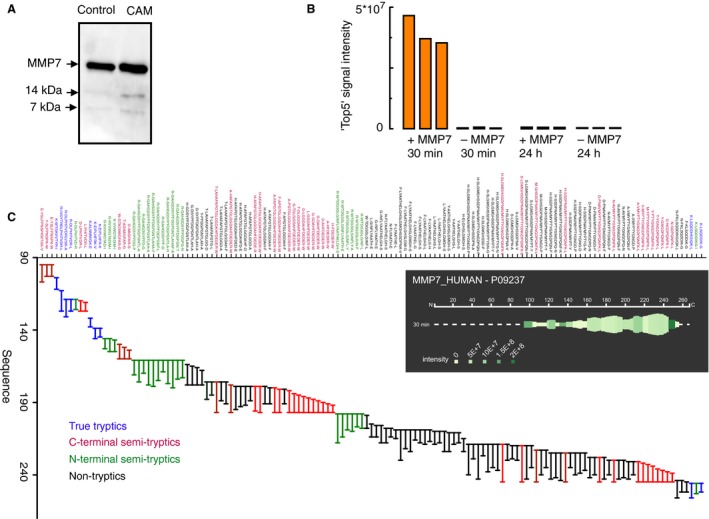
Proteolysis of MMP‐7 by myofibroblasts. (A) In the medium of myofibroblasts incubated (37°C, 30 min) with recombinant human MMP‐7 (2 *μ*g), western blot reveals both the starting material (19 kD, see control samples of MMP‐7 incubated in medium in the absence of cells, ‘Control’) and two bands corresponding to degradation products at 14 and 11 kD. (B) Label‐free quantification of MMP‐7 in the medium, based on the summed intensity of 5 unique tryptic peptides shows substantial loss of protein by 24 h compared with 30 min. (C) Analysis of MMP‐7 fragments after 30 min incubation revealed numerous fragments attributable to endopeptidase cleavage followed by aminopeptidase or carboxypeptidase trimming of the cleavage products, separately or in concert; blue, tryptic peptides; red, semi‐tryptic on the N‐terminal side of Lys or Arg; green, semi‐tryptic on the C‐terminal side of Lys or Arg; black, non‐tryptic peptides. Inset is a schematic representation of the distribution and relative abundance of cleavage products averaged over three biological replicates using *Peptigram* (Manguy et al. 2017). For each residue, a green bar is drawn if this position is covered by at least one peptide in the sample; height of the bars is proportional to the count of peptides overlapping this position; colour intensity is proportional to the summed ion intensities of peptides overlapping this position, with dark green indicating high peptide intensity and light green indicating low peptide intensity. No fragments are observed before residue 95 which is the start of the mature form of MMP‐7 used in this study.

We considered the possibility that myofibroblasts might rapidly metabolize MMP‐7. The metabolism of MMP‐7 has not been well‐studied previously, but we found by western blot that after 30 min incubation of MMP‐7 with myofibroblasts there remained intact MMP‐7 as well as minor bands corresponding to cleavage products of 11 and 14 kDa (Fig. 6A). Quantification of MMP‐7 from the abundance of 5 unique tryptic peptides indicated abundant protein at 30 min, but almost complete loss after 24 h incubation (Fig. 6B). We reasoned that cleavage products of MMP‐7 would appear as semi‐tryptic peptides after the usual processing of samples by tryptic digestion. Following incubation of myofibroblasts with MMP‐7 for 30 min, we then identified 98–130 (mean 113, in three replicates) semi‐ or non‐tryptic fragments of MMP‐7 which on alignment indicated endopeptidase cleavages particularly in the region 175‐217 (numbering from the initiator Met in preproMMP‐7), followed by carboxy‐ and aminopeptidase trimming of the cleavage products (Fig 6C). The data are compatible with progressive endopeptidase cleavage of MMP‐7 by myofibroblasts followed by extensive carboxy‐ and aminopeptidase trimming of the products.

### Functional significance of MMP7 expression in OE33 cells

**Figure 7:**
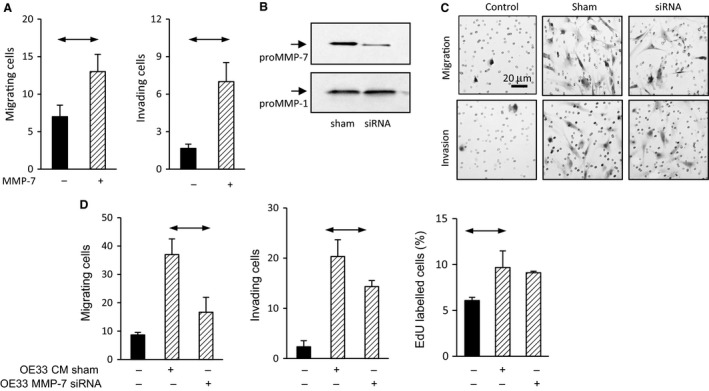
MMP‐7 mediates cancer cell stimulation of myofibroblast migration. (A) recombinant MMP‐7 (2 *μ*g/mL) stimulates the migration (left) and invasion (right) of EAC myofibroblasts in Boyden chambers. (B) siRNA knockdown of MMP‐7 expression in OE33 cells reduces expression by 80% while expression of MMP‐1 is preserved. (C) representative images of myofibroblasts in migration (top) and invasion (bottom) assays using Boyden chamber chemotaxis assays in response to conditioned medium from control (left), sham (center), and siRNA‐treated (right) OE33 cells; scale bar 20 *μ*m. (D) Conditioned medium (hatched bars) from sham and MMP‐7 siRNA‐treated OE33 cells stimulates migration (left), invasion (center), and proliferation (right) of EAC myofibroblasts and siRNA treatment significantly reduces the migration and invasion responses. Representative results are shown for migration studies in two different CAM lines. Horizontal bars, *P* < 0.05.

Because MMP‐7 is implicated in the remodeling of the microenvironment elsewhere in the upper gastrointestinal tract (e.g., stomach) (McCaig et al. 2006) we asked whether there might be analogous functions in EAC. In initial studies, we found recombinant human MMP‐7 had a modest effect in stimulating migration and invasion of OE33 cells by 1.8 and 1.5 fold, respectively. However, there was an approximately twofold stimulation of myofibroblast migration and a substantial 4‐5 fold stimulation of invasion in Boyden chamber chemotaxis assays (Fig. 7A). We then measured migration and invasion of EAC myofibroblasts in Boyden chambers in response to OE33 cell CM either with or without knockdown of MMP‐7 expression by siRNA. Western blot confirmed that following siRNA treatment there was an 80% reduction in proMMP‐7 in OE33 media, but not MMP‐1 run as a control (Fig. 7B). The OE33 cell CM significantly increased both migration and invasion of EAC CAMs but following MMP‐7 knockdown there was a significant reduction in cell migration in response to CM, and a smaller reduction in invasion (Fig. 7C and D); similar results were obtained with migration of a second EAC CAM line using OE33 cell CM with or without siRNA knockdown. Moreover, OE33 CM stimulated migration of a third EAC CAM and this was inhibited by MMP‐7 immunoneutralization (CM: 1.8 ± 0.2 fold increase in migration over control, vs. 1.0 ± 0.12 fold after immunoneutralization). Interestingly, OE33 CM also increased proliferation of myofibroblasts in serum‐free medium, but the knockdown of MMP‐7 had no effect on proliferation in these experiments (Fig. 7D).

## Discussion

This study shows expression of MMP‑7 is associated with the progressive epithelial changes that characterize the transformation of normal squamous epithelium through the preneoplastic condition of BE to EAC. Its expression is highest at the invasive front in EAC where it is also expressed in stromal spindle‐shaped cells. Although gastrin regulates MMP‐7 expression in stomach and CCK2 receptors are expressed in BE, we found no evidence that gastrin is associated with increased MMP‐7 in the progression to EAC. At least in an EAC cell line, we found high constitutive expression of proMMP‐7 was partly attributable to activation of PI 3‐kinase. There was some expression of proMMP‐7 in cultured myofibroblasts as indicated by immunocytochemistry, western blot and ELISA of cell extracts, but we were unable to detect its presence in myofibroblast medium. However, the result of studies of myofibroblast migration and invasion in response to MMP‐7 secreted by epithelial cells raises the possibility that MMP‐7 plays a role in the epithelial‐mesenchymal signaling that underpins the stromal cell reorganization characteristic of EAC.

There are two main types of esophageal cancer: EAC which predominates in western societies and squamous esophageal cancer which occurs in high incidence in Asia. There have been a number of studies of MMP‐7 in squamous esophageal cancer but MMP‐7 has been less well studied in EAC and BE (Adachi et al. 1998; Yamashita et al. 2000; Tanioka et al. 2003; Miao et al. 2015). Salmela et al., showed by in situ hybridization that MMP‐7 was abundantly expressed in epithelial cells in EAC whereas other MMPs (MMP‐1, MMP‐8) were less widely expressed and TIMP‐1 and ‐3 exhibited predominantly stromal expression (Salmela et al. 2001). Our data suggest relatively low expression in normal squamous esophageal epithelium and progressively increased expression in BE, LGD, HGD, and EAC with high expression at the invasive front where both cancer and stromal cells express MMP‐7. A previous study using in situ hybridization noted MMP‐7 expression in epithelial cells, while it was normally absent in stromal cells. The difference in detecting in MMP‐7 in stromal cells by immunohistochemistry compared with in situ hybridization (Salmela et al. 2001) may conceivably reflect different sensitivities of the two methods. Regardless of the functional significance of MMP‐7 in EAC myofibroblasts we suggest that its pattern of expression may provide a useful marker of invasive activity in these tumors.

Because myofibroblasts play important roles in defining the cancer microenvironment and cancer progression we examined the biology of MMP‐7 in myofibroblasts in more detail. We screened three different EAC myofibroblast lines and found evidence of expression in cell extracts but no evidence of presence in the medium. The localization of MMP‐7 activity to vesicles and the increased abundance after BFA treatment are compatible with the idea that proMMP‐7 is localized to the secretory pathway. It is conceivable that following secretion by myofibroblasts there was rapid degradation to fragments that were undetectable by the antibody (N‐terminal specific) used for western blot and ELISA. Because relatively little is known of MMP‐7 metabolism in any tissue, we undertook proteomic studies to determine the pattern of cleavage of recombinant MMP‐7 by myofibroblasts. The data suggest endopeptidase cleavage of MMP‐7 particularly in the mid‐ and C‐terminal part of the protein in myofibroblast medium followed by amino‐ and carboxypeptidase trimming of the products. At 30 min of incubation of MMP‐7 with myofibroblasts there were minor bands seen on western blots that were in reasonable agreement with predicted N‐terminal fragments of MMP‐7 following endopeptidase cleavage (9.5 and 13.5 kD). We suggest that the low abundance of MMP‐7 in myofibroblast medium is likely to be attributable both to low rates of secretion and an extensive capacity for degradation. In the future it will be useful to repeat these studies using recombinant proMMP‐7.

It has been known for some time that CCK2R, at which the antral hormone gastrin acts, is expressed in BE and quite recently CCK2R has been localized to a LGR5+ stem cell population (Haigh et al. 2003; Lee et al. 2017). Barrett's esophagus patients are typically treated with proton pump inhibitors to suppress acid secretion; however, inhibition of acid secretion also removes an inhibitory effect of acid on the G‐cell leading to increased circulating gastrin (Lamberts et al. 1993). Because gastrin stimulates MMP‐7 expression in stomach (Varro et al. 2007) we hypothesized that it had similar effects in BE and EAC. Using cells expressing the CCK2R (i.e., OE33‐Gr cells) we were readily able to confirm receptor expression and demonstrate the capacity of cells to respond to gastrin by an increase in intracellular calcium. However, gastrin had no effect on MMP‐7 expression, neither did stimulation of protein kinase C (which is a downstream mediator of CCK2R) using PMA. The so‐called non‐classical gastrins (Gly‐Gastrin, G17‐CFP and progastrin) that have been ascribed roles in cancer progression, including EAC, were also inactive (Dockray 2004; Ogunwobi and Beales 2008; Beales and Ogunwobi 2009). Moreover, we found no evidence that MMP‐7 transcript abundance in BE biopsies varied with serum gastrin. Unexpectedly, however, we found an inverse relationship between CCK2R abundance and increasing serum gastrin concentrations. The latter data therefore suggest a previously unsuspected auto‐regulation of CCK2R expression in BE and raise the possibility that the effects of elevated circulating gastrin are offset by decreased CCK2R expression. Thus, we suggest that mechanisms other than increased serum gastrin are responsible for the increased MMP‐7 that occurs in BE and EAC.

Activation of both the MAPkinase and PI 3‐kinase pathways has been described in BE and EAC together with evidence of a link to increased cell proliferation and expression of proteins involved in migration and invasion including MMPs (Vona‐Davis et al. 2005; Phillips et al. 2006; Keld et al. 2010; Saeed et al. 2016). A role for the MAPkinase pathway has also previously been reported for stimulation of MMP‐1 expression in OE33 cells (Keld et al. 2010). However, in our studies an inhibitor of p42/44 MAPkinase activation did not supress MMP‐7 expression. In the case of the PI 3‐kinase pathway, the downstream mediator Akt is present in its activated (phosphorylated) state in BE, dysplasia and EAC (Beales et al. 2007) and it is known that OE33 cells over‐express Akt while the PI 3‐kinase inhibitor LY294002 suppressed stimulated proliferation (Beales et al. 2007; Pal et al. 2012). The present data indicate that LY294002 also significantly reduced the abundance of proMMP‐7 in medium and cell extracts compatible with inhibition of expression. In view of the continued interest in targeting PI 3‐kinases in cancer (Yap et al. 2008; Fruman and Rommel 2014) we therefore suggest that MMP‐7 may have potential both as downstream target of this pathway in BE/EAC and as a biomarker of it. Nevertheless, care is required in interpretation of the data not least because some other related cells lines including for example FLO‐1 cells and the gastroesophageal junction cancer cell line OE19 express MMP‐7 either weakly or not at all (Keld et al. 2010).

**Figure 8:**
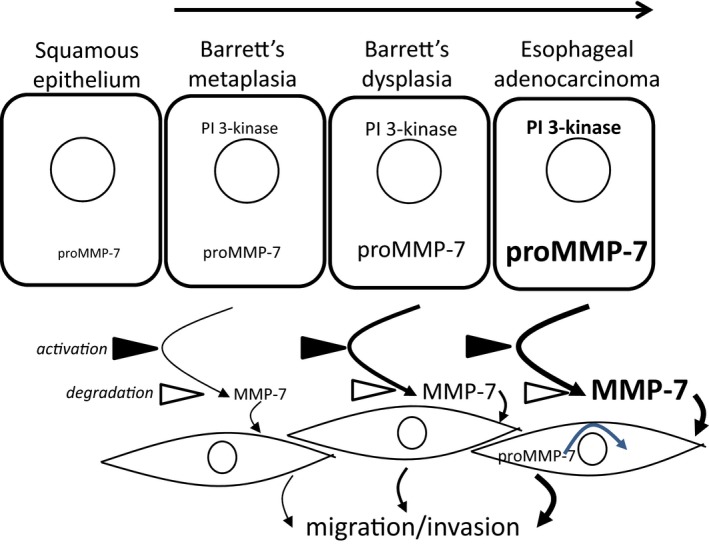
Schematic representation of the role of MMP‐7 in the progression from Barrett's esophagus to esophageal adenocarcinoma. Differences in font size and line thickness indicate the magnitude of effects. It is suggested that there is progressively increased expression of proMMP‐7 in progress to EAC at least in part due to activation of PI 3‐kinase, that following secretion conversion of proMMP‐7 to MMP‐7 activates stromal cells stimulating myofibroblast migration and invasion and remodeling of the cancer cell microenvironment.

Several MMPs have previously been shown to be increased in esophageal adenocarcinoma, including MMP‐1 (Grimm et al. 2010), MMP‐9 and MMP‐13 (Davelaar et al. 2015). Increased MMP activity detected by MMPsense680 activity occurs in the progression to EAC; two of the main activators of this substrate are MMP‐9 and MMP‐13, the former is low in relative abundance in metaplasia and increases in EAC, the latter is higher in metaplasia than EAC indicating potential switches in MMP expression in progression (Davelaar et al. 2015). The present study indicates that MMP‐7 should also be included as a functionally relevant MMP that is implicated in the progression to EAC. Since MMP‐7 stimulates esophageal myofibroblast migration and invasion and was partly responsible for the effect of OE33 cell CM in stimulating myofibroblast responses we suggest that the increased MMP‐7 in BE and EAC contributes to a microenvironment favoring tumor invasion both by degradation of extracellular matrix and by remodeling stromal cell distribution by control of myofibroblast motility (Fig 8). In this sense, there may be parallels between the role of MMP‐7 in shaping the esophageal microenvironment and that in the stomach and colon where there is already evidence that MMP‐7 stimulates stromal cell function via cleavage of insulin‐like growth factor binding proteins (IGFBPs) leading to increased bioavailability of IGF‐I and –II (Hemers et al. 2005; McCaig et al. 2006).

In conclusion, the present data support the concept that MMP‐7 has potential both as a target that drives EAC progression and as a biomarker of it. Functionally, the role of MMP‐7 in promoting invasion that has previously been recognized in other gastrointestinal cancers (Adachi et al. 2009) is also likely to apply to EAC. In addition, the unexpected expression of MMP‐7 in myofibroblasts associated with invasive EAC may be useful as a novel dimension in histopathology even though the functional significance in this case remains to be determined.

## Conflicts of Interest

The authors disclose no conflicts.
